# The Death of King Edward VII

**Published:** 1910-06-04

**Authors:** 


### News and Events of the Week.

### THE DEATH OF KING EDWARD VII.

The following loyal addresses were passed at a special meeting of the Royal Infirmary of Edinburgh, held last week: "To the King's Most Excellent Majesty,—May it please your Majesty, We, the Members of the Board of Management of the Royal Infirmary of Edinburgh, beg leave to assure your Majesty, on your Accession to the Throne, of our devoted loyalty to your Majesty's Person and Throne. We humbly offer to your Majesty the expression of our heartfelt sympathy with your Majesty and your Royal House in the grievous loss which you have sustained through the demise of your honoured and revered father, our late most gracious Sovereign. While we mourn with our fellow-subjects throughout your Majesty's wide dominions the death of a Monarch who was distinguished for his unswerving devotion to the duties of his exalted position, for his high sense of responsibility, and for the noble and successful efforts he made to promote alike the best welfare of the people over whom he ruled, and the peace of the whole world, we do not forget that our late King took a peculiarly deep interest in the work of hospitals and kindred institutions for the mitigation and relief of disease and suffering. We rejoice to know that your Majesty has likewise taken a leading part in promoting the greater efficiency of the hospitals of your country, and we feel sure that their beneficent aims will always lie near to your heart. On three occasions your Majesty has honoured this infirmary with a visit, and we shall ever remember with heartfelt gratitude the appreciation and sympathy you manifested for its work. We fervently pray that your Majesty may long be spared to rule over a united and devoted people, and that Almighty God may grant you a happy and prosperous reign."

To Her Gracious Majesty Alexandra, the Queen Mother: "May it please your Majesty, We, the Members of the Board of Management of the Royal Infirmary of Edinburgh, beg leave to tender to your Majesty our respectful and heartfelt sympathy in the grievous bereavement which has befallen you through the death of your august husband, King Edward, of gracious and blessed memory. Your Majesty's most touching message to the nation has filled all our hearts with a feeling of profoundest veneration for the Queen, who has ever held the love of the people of this her adopted country, since first she landed on its shores. We recognise in the simple dignity of the words in which your Majesty reveals the depth of your personal sorrow the true heart of one who has shed lustre on a Throne by her womanly virtues as wife and mother; and your moving appeal for the prayers of the nation to comfort and sustain you in your affliction will meet with an earnest response in every home made sacred by ties of family love. We would assure your Majesty that the loss which falls so heavily upon your Royal Home is felt acutely by us all, and not least so by those who are associated in the beneficent work of succouring the suffering poor. Our late most gracious King, distinguished as he was for his devotion to the things which make for peace, and for the matchless influence he brought to bear in establishing friendly relations among the nations of the civilised world, was yet a true father to his own people, to whom he gave unstinted service—showing to the very last an intense personal interest in all that concerned their welfare and their happiness. More especially he proved himself a friend indeed to the victims of pain and disease. The sacred work carried on in the hospitals and infirmaries throughout his realms ever found in him a most generous patron and zealous helper. The managers of this infirmary recall with deepest gratitude the many favours he extended to it, and the warm interest he manifested in its affairs. As sharers in a measure in your Majesty's sorrow, we offer you our humble condolence, praying that the comfort which the God of all consolation alone can give may rest upon you abundantly, and upon all the members of your illustrious family."

---

On the sad and memorable occasion of the funeral of his late Majesty King Edward VII. the chairman and governors of St. George's Hospital (of which hospital his late Majesty was Patron), having, in respectful and grateful remembrance of the gracious and active interest which his late Majesty, as Founder of the King's Hospital Fund and as Patron of a hundred and more hospitals, was pleased to take in all hospitals and medical charities throughout his Kingdom, and of St. George's and all other London hospitals in particular, as a slight and humble token of respect to the late King and to mark their loyal sympathy with his Gracious Majesty the King, decided to invite representatives of the larger voluntary London hospitals to be present at Hyde Park Corner during the procession and for the memorial service in the hospital chapel after, the wish of the chairman and governors being to make some small public expression of the great loss which they and all hospitals feel they have sustained in the death of his late Majesty, who was ever untiring in his interest and ready with his help in all things connected with the medical charities.

The following accepted the invitation to be present: From Atkinson Morley's Hospital: Miss Sharp (matron), Miss Baylis (sister), Miss Richards (nurse); King's College Hospital: Capt. H. S. Tunnard, Miss Brown, Miss Bredin, and Miss Todd; Royal Free Hospital: Conrad W. Thies, Esq., R. H. Gamlen, Esq., Miss Cox Davies (matron), Miss Wills, and Miss K. M. Read; Middlesex Hospital: F. Clare Melhado, Esq., Colonel the Hon. C. T. Gathorne Hardy, Miss A. Lloyd Still (matron), Miss E. G. Morgan, and Miss V. Daunt; Guy's Hospital: Hy. Williams, Esq., A. H. Campbell, Esq., Miss Haradine (assistant matron), Miss Strick (superintendent private nursing staff), Miss Cross (sister); St. Thomas's Hospital: G. Q. Roberts, Esq., J. G. Wainwright, Esq., Miss Hamilton (matron), Miss L. Smith, and Miss E. Flemming; St. George's Hospital: G. E. Friend, Esq., A. Wm. West, Esq., Miss McCall-Anderson, R.R.C. (matron), Miss Brown Watson (assistant matron), and Miss Allen (sister); St. Bartholomew's Hospital: Patrick L. Blyth, Esq., Thos. Hayes, Esq., Miss Cutler (matron), Miss Skillman (sister), and Miss Munro (sister); Westminster Hospital: Sir John Wolfe Barry, K.C.B., S. M. Quennall, Esq., Miss Cave (matron), Miss J. Clarke (sister), and Miss Farrington (sister); London Hospital: Miss Hosking, Miss Janet Scott, and Miss Apperley; Charing Cross Hospital: The Rev. A. W. Oxford, M.D., Walter Alvey, Esq., Miss Heather Bigge (matron), Miss Pike (assistant matron), and Sister Alexandra; University College Hospital: H. Batty-Shaw, Esq., M.D., J. Gerald Buckle, Esq., Miss Finch (matron), Miss Riddell, and Miss Steele; St. Mary's Hospital: W. Austen Leigh, Esq., Miss Davies (matron), Miss Morrison, and Miss Colbecke; Hospital Saturday Fund: A. W. Davis, Esq.; Hospital Sunday Fund: Sir Edmund Hay Currie.

---

A special meeting of the Council of the Metropolitan Hospital Sunday Fund was held last week at the Mansion House, the Lord Mayor being in the chair, when the Archdeacon of London proposed a resolution of sympathy with the King, from which the following is taken: "The Council remember with the deepest gratitude that on the occasion of His late Majesty's visit to St. Paul's Cathedral on the thirty-first Hospital Sunday in 1903, accompanied by his gracious Consort and many of the Royal Family, the collections for the Fund in places of worship reached a record total; and that under the gracious patronage of the Sovereign the Fund has, since its inception in 1872, collected no less that £1,680,000 for the hospitals and medical charities of London, in which His late Majesty's interest was deep and lifelong." Sir J. Bell seconded the resolution, which was adopted.

---

At a meeting of the Committee of Management of the Seamen's Hospital Society, which was held last month, the Chairman rose to record the death of the Society's patron, King Edward VII., and in doing so expressed in sympathetic terms the deep sorrow of the Committee of Management of the Seamen's Hospital Society, and declared the irreparable loss which the hospitals and the nation has sustained. The Chairman dwelt upon the great services rendered by His late Majesty to the hospitals in general in the establishment of King Edward's Hospital Fund, and stated that it was gratifying to the Society to know that in arranging the details of the Fund His Majesty had availed himself largely of the assistance of Sir Henry Burdett, a Vice-President and former Secretary of the Society. The Chairman concluded by moving that the deep sorrow of this Committee be recorded on the minutes, and that a vote of condolence on the death of King Edward be forwarded to the proper quarter. Captain A. W. Clarke also expressed the sorrow of the Committee, and seconded the above resolution, which was carried unanimously.

---

Sir Thomas Barlow, R.C.P., it is understood, has been nominated President of the International Medical Congress, which meets in London in 1913, and Dr. W. P. Herringham to be its general secretary. The nominations have yet to be confirmed.

---

The second summer school of the North Wales Temperance Federation will be held in the Higher Grade Schools, Colwyn Bay, from July 25 to August 6, 1910. The course consists of practical and theoretical classes in temperance, hygiene, physical training, and Nature-study (with field excursions). Fee, for the whole or a part of the course, 5s. For further particulars apply to the Secretary, Rev. J. Glyn Davies, 5 Church Street, Rhyl.

---

The annual general meeting of the Research Defence Society will be held on Friday, June 3, at 5 o'clock, by kind permission, in the Library of the Royal College of Physicians, Pall Mall East, S.W. The chair will be taken by the Right Hon. the Earl of Cromer, president of the Society. The other speakers will be Sir Richard Douglas Powell, Bart., K.C.V.O., Sir David Bruce, K.C.B., F.R.S., Mr. Anthony Hope Hawkins, and Mrs. Scharlieb, M.D.

---

A special course of six demonstrations on the surgery of the nervous system will be given at the National Hospital for the Paralysed and Epileptic, Queen Square, Bloomsbury, W.C., on Fridays, June 3, 10, and 17, and Wednesdays, June 8, 15, and 22, at 5 o'clock, by Mr. Percy Sargent. The syllabus is as follows: Friday, June 3.—(1) Applied anatomy: Brain and spinal cord. Wednesday, June 8.—(2) Infective diseases: Sinus thrombosis, extra-dural abscess, cerebral and cerebellar abscess, encephalitis, meningitis. Friday, June 10.—(3) Intracranial tumours. Wednesday, June 15.—(4) Cranial injuries. Friday, June 17.—(5) Surgical treatment of lesions of the spinal cord. Wednesday, June 22.—(6) Peripheral nerves. The fee for this course will be one guinea. Cards for admission may be obtained from the Secretary, at the hospital, to whom all fees are payable.

---

In one of the articles on the late King which Mr. D. Williamson is contributing to a contemporary occurs the following:—

An eminent surgeon told me the circumstances under which the King conferred knighthood upon him. The doctor had earned the distinction by long practice of his profession and by his personal contact with the Court. But when the knighthood was gazetted it happened that he was abroad on a holiday tour. On his return, a day was appointed for attendance at Buckingham Palace. On the previous day a note was sent to him, by the King's command, saying that he need not wear Court dress, as the King was aware that the surgeon was due at the hospital, and would find it inconvenient. That considerateness of the King was followed by another charming act. After His Majesty had conferred knighthood he said to the surgeon, "Now, I don't want you to think of retiring from active work because of this honour. For our sakes, as well as for your other patients, I hope you will continue your practice." Could there have been a happier compliment?

The annual meeting of the Factory Girls' Country Holiday Fund will be held at Aubrey House, Aubrey Road, Campden Hill, W. (by kind permission of W. Cleverley Alexander, Esq.), on Wednesday, June 15, at 5 P.M. Chairman, the Lord Bishop of Kensington.

Messrs. Williams and Norgate announce a new quarterly review of medical jurisprudence—*Archives Internationales de Médecine légale*, published under the auspices of the Belgian Society of Legal Medicine. The collaborators are drawn from almost all parts of the Continent. The editors' preliminary notice (which is in very Frenchified English, just as, probably, most French speeches by Englishmen are highly Anglicised) states that the only previous venture of the kind was by a German, but that his undertaking failed; perhaps because he included in his review papers on hygiene, and only published it annually, and moreover referred solely to German authors. The last fault is certainly one that Germans have been guilty of before now, although they might make the excuse that in mass, and on the whole, in quality, of scientific work, their country leads the world. The present venture will in the one part print original articles in the four congress languages, and devote the other to analysis or summaries of noteworthy literature. These summaries are printed on numbered removable sheets. The subscription is 20 francs a year.

Part IV. of the *Treasury of Human Inheritance*, issued by the Francis Galton Laboratory for National Eugenics, has just appeared. It contains sections on hare-lip and cleft-palate by Dr. Rischbieth, on congenital cataract by Mr. Bishop Harman, and on deaf mutism. There are seven plates of illustrations and nine of pedigrees. Published by Messrs. Dulau and Co., the work maintains to the full its very high standard, and we are sorry to see that the high cost of its production has compelled Professor Pearson to appeal for more subscribers and support.

The Animal Defence and Anti-Vivisection Society states that it did not issue or distribute the leaflet connecting King Edward's death with vaccine treatment.

Mr. A. J. Crane has been appointed to the Finance Committee and Mr. W. J. Lancaster to the Emergency Committee of the Bromsgrove, Droitwich, and Redditch Hospital Committee.

Dr. William Hoffmeister, M.V.O., of Cowes, who was surgeon apothecary to Queen Victoria and the Royal Family at Osborne, has announced his retirement from practice. His many appointments include those of surgeon to the Royal Yacht Squadron, M.O.H. to the Cowes District Council (since 1875), Cowes Port medical officer, Union medical officer of the Cowes District of the Isle of Wight. For nearly 44 years Dr. Hoffmeister has been public vaccinator and surgeon to the Cowes Oddfellows, and certifying surgeon under the Factory Acts.

The architectural portions of the Shakespeare Memorial in Southwark Cathedral can be begun as soon as a further £240 have been subscribed. Subscriptions will be acknowledged by the Hon. Secretary, Dr. R. W. Leftwich, M.D., 125 Kennington Park Road, S.E.

At the meeting of the Farnham Rural District Council last week the medical officer reported that cases of appendicitis in the district were increasing alarmingly. He thought that the cause was the excessive use of preservatives which disguised the presence of decomposed food. He had seen 30 cases of appendicitis that year. The cases occurred at all ages. It was decided to send a copy of the report to the Local Government Board.

The Psycho-Therapeutic Society has removed from 3 Bayley Street, Bedford Square, to 34 Bloomsbury Square, London, W.C.

The Harben Lectures of the Royal Institute of Public Health will be delivered by Brevet Lieut.-Col. Sir W. B. Leishman, M.B., F.R.S., Professor of Pathology in the Royal Army Medical College, London, in the Lecture Room of the Institute, on Wednesdays, June 8, 15 and 22, at 6 P.M. Subject: "Anti-Typhoid Inoculation." Fellows and members, and all others interested, are cordially invited to attend.

At an Examination in Sanitary Science as applied to Buildings and Public Works, held in London, on May 19 and 21, 1910, by the Royal Sanitary Institute, twenty candidates presented themselves, and eight were granted certificates. At an Examination in Hygiene in its bearing on School Life, held in London, on the same dates, nine candidates presented themselves and five were granted certificates, and at an Examination for Women Health Visitors and School Nurses, forty-five candidates presented themselves, and twenty-seven were granted certificates. At an Examination for Inspectors of Nuisances, eighty-eight candidates presented themselves, and forty-eight candidates were certified, as regards their Sanitary Knowledge, competent to discharge the duties of Inspector of Nuisances under the Public Health Act, 1875.

The following resolution was passed unanimously at the conference of representatives of the County Nursing Associations and of the Council of Queen Victoria's Jubilee Institute, which met last week to discuss, among other questions, the usual practice as to payment of the doctor's fee where he had been called in at the request of a nurse to a case, other than one of parish relief, in which the doctor had great difficulty in recovering his fee: "That the Institute be asked to organise a deputation to the Lord President of the Council pointing out the importance of providing for the payment of the doctor's fee in midwifery cases and pressing for such amendment as will secure the payment of the fee." On the question of obtaining midwifery scholarships for Queen's nurses it was agreed that county councils generally be asked whether an extension of the facilities for midwifery training could be made to include Queen's nurses as well as less highly trained women.

Mr. C. T. Kingzett, F.I.C., F.C.S. (the Chairman), presided at the annual meeting of the "Sanitas" Company, Ltd., held at the Works, Limehouse, on the 25th ult., and congratulated the shareholders upon the continued prosperity of the undertaking, stating that, notwithstanding the most unfavourable summer of 1909, the past year had been a record one both in respect of business and profit. Attention was directed to the growing sales of the various "Sanitas" fluids and powders, and to a recent report issued by the *Lancet* with respect to coal tar disinfectants, which showed that "Sanitas-Bactox" is "the best and cheapest" of all the homogeneous disinfectants which were examined by that Commission, and that "Sanitas-Okol" takes first place amongst disinfectants of the ready-made emulsion order. A dividend and bonus were declared, making, with the interim dividend already paid, a total distribution of 7½ per cent. for the year, and £2,000 was placed to reserve. There was also initiated a profit-sharing system in which all the employees of the company participate according to their wages and the length of their services to the company.

